# A preliminary report on the distribution of lizards in Qatar

**DOI:** 10.3897/zookeys.373.5994

**Published:** 2014-01-23

**Authors:** Dan Cogălniceanu, Aurora M Castilla, Aitor Valdeón, Alberto Gosá, Noora Al-Jaidah, Ali Alkuwary, Essam O. H. Saifelnasr, Paloma Mas-Peinado, Renee Richer, Ahmad Amer Mohd Al-Hemaidi

**Affiliations:** 1 University Ovidius Constanţa, Faculty of Natural Sciences and Agricultural Sciences, Al. Universitatii 1, corp B, 900740 Constanţa, Romania; 2 Department of Biodiversity, Qatar Environment and Energy Research Institute (QEERI), Qatar Foundation, Education City, P.O. Box 5825, Doha, Qatar; 3 Forest Sciences Centre of Catalonia (CTFC), Road Sant Llorenç de Morunys km2, 25280 Solsona, Catalonia, Spain; 4 Department of Herpetology, Aranzadi Society of Sciences. Zorroagagaina, 11. San Sebastián, Spain; 5 Department of Geography and Regional Planning. University of Zaragoza. Pedro Cerbuna, 12. Zaragoza, Spain; 6 Wildlife Research Section, Ministry of Environment, Qatar, P.O. Box 7635, Doha, Qatar; 7 Agricultural Research Center (ARC), Ministry of Agriculture, Egypt; 8 Genetic Resources Department, Biotechnology Centre, Ministry of Environment, Qatar, P.O. Box 200022, Doha, Qatar; 9 Department of Biodiversity and Evolutionary Biology; National Museum of Natural Sciences; Spanish National Research Council (CSIC); C/ José Gutiérrez Abascal 2, 28006 Madrid, Spain; 10 Weill Cornell Medical College, Qatar Foundation, Education City, P.O. Box 5825, Doha, Qatar; 11 Ministry of Environment, Qatar, P.O. Box 7635, Doha, Qatar

**Keywords:** Reptilia, geographic distribution, species richness, inventory, maps, biodiversity, atlas

## Abstract

We have updated the list of the lizard species present in Qatar and produced the first distribution maps based on two field surveys in 2012 and 2013. We used the QND95/Qatar National Grid with a grid of 10 × 10 km squares for mapping. Our results show the occurrence of 21 lizard species in Qatar, from the 15 species indicated in the last biodiversity report conducted in 2004. The most abundant family found in Qatar is Gekkonidae with nine species (*Bunopus tuberculatus*, *Cyrtopodion scabrum*, *Hemidactylus robustus*, *H. flaviviridis*, *H. persicus*, *Stenodactylus arabicus*, *S. slevini*, *S. doriae*, *Pseudoceramodactylus khobarensis*), followed by Lacertidae with four species (*Acanthodactylus schmidti*, *A. opheodurus*, *Mesalina brevirostris*, *M. adramitana*), Agamidae with three species (*Trapelus flavimaculatus*, *Uromastyx aegyptia*, *Phrynocephalus arabicus*), Scincidae with two species (*Scincus mitranus*, *Trachylepis septemtaeniata*), and Varanidae (*Varanus griseus*), Sphaerodactylidae (*Pristurus rupestris*) and Trogonophiidae (*Diplometopon zarudnyi*) with one species each. The species richness fluctuated largely across Qatar between one and eleven species per grid square. We believe that the lizard fauna records in Qatar are still incomplete and that additional studies are required. However, our study here fills a gap concerning lizard biodiversity knowledge in the Gulf Region.

## Introduction

The rapid worldwide decline of reptiles has raised concerns about their conservation and the urgent need for action (Gibbons et al. 2000). A recent survey of the status of reptiles has shown that nearly one of five reptilian species are threatened with extinction and one of five classified as Data Deficient (Böhm et al. 2013). The decline of reptiles has been influenced by a variety of threats such as habitat loss, degradation and fragmentation, pet trade, invasive species, pollution, diseases and climate change (Böhm et al. 2013, Cox and Temple 2009, Gibbons et al. 2000). For the management and conservation of reptiles, quality species and population data is required to understand and predict the potential impacts caused by human activities. Because the lack of occurrence data is limiting both our understanding of the species needs and the management options (Primack 2010), several global scale initiatives were initiated to compile the vast biodiversity datasets (e.g. Global Biodiversity Information Facility – GBIF, Encyclopedia Of Life – EOL). Such databases accompanied by the advances in computation and advanced analysis allow for the proper management of data based on scientific knowledge (Matin et al. 2012, Reese et al. 2005). Unfortunately, the data available in these global databases is spatially biased, mainly because only few countries are contributing the majority of data.

**Figure 1.**
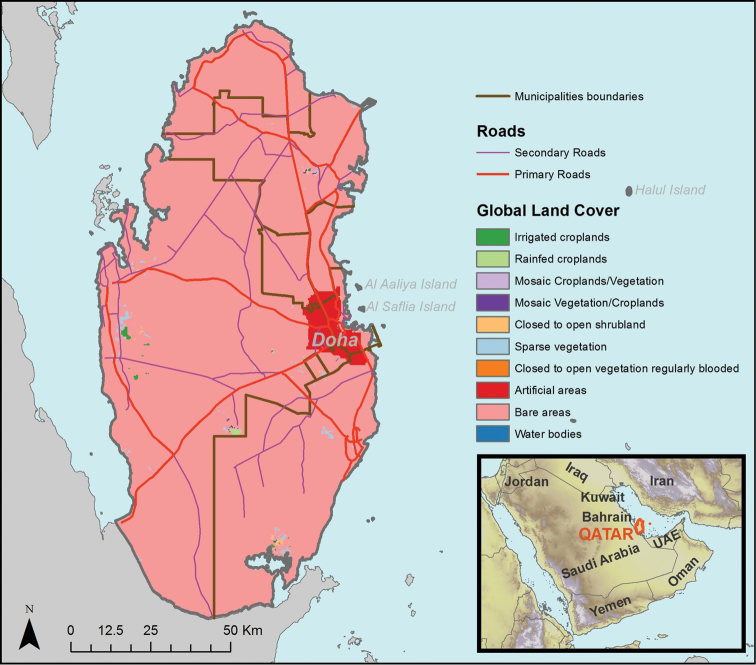
Location of Qatar within the Arabian Peninsula, and general map of Qatar. Global Land Cover was obtained from GlobCover 2.3 (ESA 2010)

The state of Qatar is a peninsula with an area of 11,571 km^2^ that lies between 24–27°N and 50–52°E (Figure 1). The peninsula projects 186 km north from Saudi Arabia and varies in width between 55 and 90 km. Much of the country is flat with a highest elevation of 103m. Global Land Cover v2.3 identifies 10 coverage categories in Qatar (Figure 1, ESA 2010). The soil consists of a low barren plain covered with rocks, sand or small dunes, arable land representing only 1.6% (Hutchinson Encyclopedia 2011). Qatar’s climate is hot and arid with an average annual mean temperature of 27 °C and 75 mm rainfall/year. There are no forests in Qatar and vegetation coverage is scarce with bushes and grasses of sporadic species and some *Acacia* trees (Batanouny 1981). Date palms and exotic vegetation is concentrated in farms across the country. Qatar has the highest density of camels in the Middle East (Richer 2008), however conservation measurements related to livestock management have been only recently implemented by the Ministry of the Environment (MOE) in Qatar. Qatar’s population has been increasing rapidly throughout the last decades, reaching currently 1.8 million inhabitants mainly concentrated (80%) in the capital of the country (Doha) (Qatar Statistics Authority 2013). The economic growth of Qatar is closely related to ongoing increases of hydrocarbon extraction, since Qatar lies in a strategic location with major petroleum and gas deposits (Qatar Statistics Authority 2013). Human impact, including oil and gas extraction, population growth and infrastructure development represent major threats for the survival of the sparsely and locally disseminated lizard populations, usually with low densities. As a Party of the Convention of Biological Diversity (CBD), Qatar has prepared a National Biodiversity Strategy and Action Plan clearly indicating the commitment to give priority to environmental issues towards sustainable development and preservation of biodiversity (MOE 2004).

The lizard fauna of Qatar is poorly known. Apart from the study of Mohammed (1988) who reports the presence of 16 lizard species in Qatar, there is no comprehensive study available for the country. Additional information reporting the presence of lizards in the country is dispersed in several scientific publications (e.g., Arnold 1980a, 1980b, Leviton et al. 1992, Castilla et al. 2011a, Metallinou et al. 2012) or the data is scattered in grey literature (Anonymous 2010, Nasher et al. 2009). To date there is not a single distribution map for any lizard species in Qatar, and very little is known about the biology and ecology of any of the Qatar lizard species except for some recent studies (Castilla et al. 2011a, b, Castilla et al. 2013, Martín et al. 2012, Herrel et al. 2013, Valdeón et al. 2013a). The aim of the present study is (i) to provide an inventory of the lizard species present in Qatar, (ii) to map their distribution, and (iii) to conduct a preliminary analysis of spatial lizard richness.

## Methods

### Mapping species occurrences

The inventory of lizard species present in Qatar was conducted during 45 days of field work in October 2012 (15 days) and in March-May 2013 (30 days), with an average time of 6 hours per day spent searching for lizards (range 3–10 hours/day). The surveys were conducted in the mainland and in three islands. Halul Island (25.67N, 52.40E) is in the E of Qatar at 81.5 km from the nearest coast in Al-Khor. Al Aaliya Island (25.41N, 51.56E) is situated at 2.5 km from the Eastern coast near Doha city. Al Saflia Island (25.34N, 51.58E) is also in the Eastern coast at a distance of 3.1 km from Doha. Al Aaliya and Al Saflia islands are separated by 5.5 km. The methods used for the inventory varied according to the habitat and time of the day, and are consistent with McDiarmid et al. (2012). The most used methods were active search during the day and night-torch surveys along transects. Most types of habitats were inventoried and special focus was given to searching under natural cover (rock-flipping) and artificial (i.e. litter) cover that often provided shelter to reptiles. On low traffic roads we conducted road surveys, both during the day and night. We occasionally also used pitfall traps and artificial cover (i.e. cardboard) for short periods of time of less than 48 hrs. The geographic location of each individual was taken on a Global Positioning System (GPS). When several individuals of the same species occurred within a short distance of one another, only one spatial data point for that given species was considered. This explains the difference between the number of species sightings and the higher number of lizards observed.

The majority of the data included in the final distribution maps come from our own surveys. However, we have also added three records from publications of Qatar University (Anonymous 2010, Nasher et al. 2009), and 41 records from local volunteers and photographers that accompanied their observations with clear photographs, GPS coordinates or grid square locations. The lizard species were identified based on morphological traits described in Arnold (1986) and Leviton et al. (1992). The most current changes in the nomenclature of the species were according to Fujita and Papenfuss (2011), Moravec et al. (2006), Pyron et al. (2013) and Bauer et al. (2013). Lizard voucher specimens are deposited in the scientific collections of the Ministry of Environment in Qatar. The distribution data belongs to the Ministry of Environment of the State of Qatar and will be uploaded to GBIF in the future.

### Data management and analysis

To make the distribution maps we proceeded as follows. The GPS geocoordinates were exported to ArcGIS 10 (ESRI) to create a shapefile, which was projected to the official reference system in the country, QND95/Qatar National Grid (UPDA 2009). A regular grid with squares of 10 × 10 km was made following the Qatar National Grid, while adapting the traditional nomenclature of UTM (Universal Transversal Mercator) or MGRS (Military Grid Reference System) squares (NGA 2013) to the Qatar National Grid (Valdeón et al. 2013b). A similar spatial resolution of 100 km^2^ was previously used in several national and regional herpetological atlases (e.g. Arnold 1995; Godinho et al. 1999; Gosá and Bergerandi 1994; Oldham and Weller 2000; Pickard and Towns 1988; Pleguezuelos et al. 2002).

Species richness per square was calculated as the number of species detected in each 10 × 10 km square. We used two relative measures as estimators of species abundance: (i) The percentage of daily sightings, measured as the number of days a certain species was observed from the total number of fieldwork days (n = 45), so we did not consider multiple sightings of the same species. (ii) The percentage of overall sightings. This was calculated as the number of times a species was observed from the total number of sightings (n = 617). We used presence-absence data for computing a species accumulation curve (SAC) and five non-parametric estimators of species richness (ICE, Chao 2, Jackknife 1, Bootstrap and Michaelis-Menten) using EstimateS 9 (Colwell 2013).

## Results

During the 45 days of field surveys we observed a total of 865 individual lizards ranging from 5 to 35 per day (average of 19 individuals/day). The total number of species sightings is lower (617), since for some species several individuals were located close together, usually under the same shelter.

**Figure 2.**
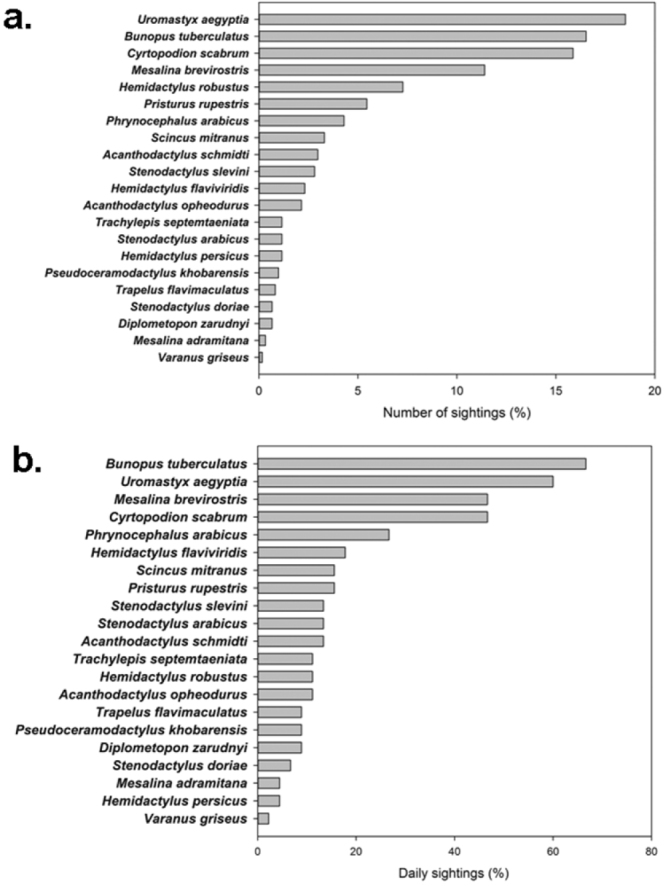
Estimates of lizard species abundance based on **a** the proportion of sightings of a certain species from the total number of sightings (n = 617), and **b** the presence of a species per day from the total number of fieldwork days (n = 45) (see methods for details).

**Figure 3.**
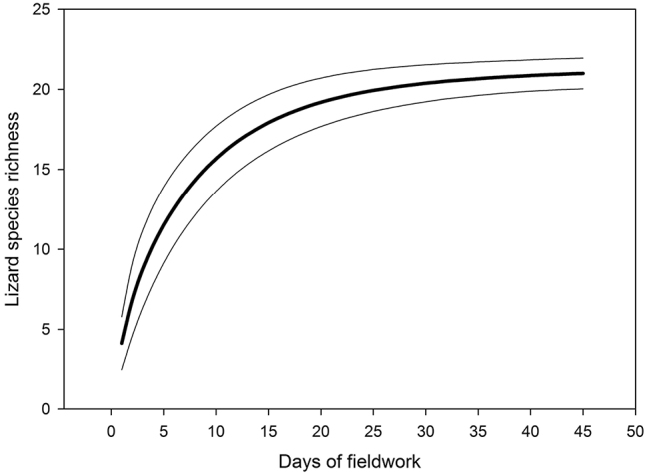
Species accumulation curve (bold line) reflecting lizard species richness based on presence-absence data for the whole country. The thin lines indicate the estimated error margins (95%).

**Figure 4.**
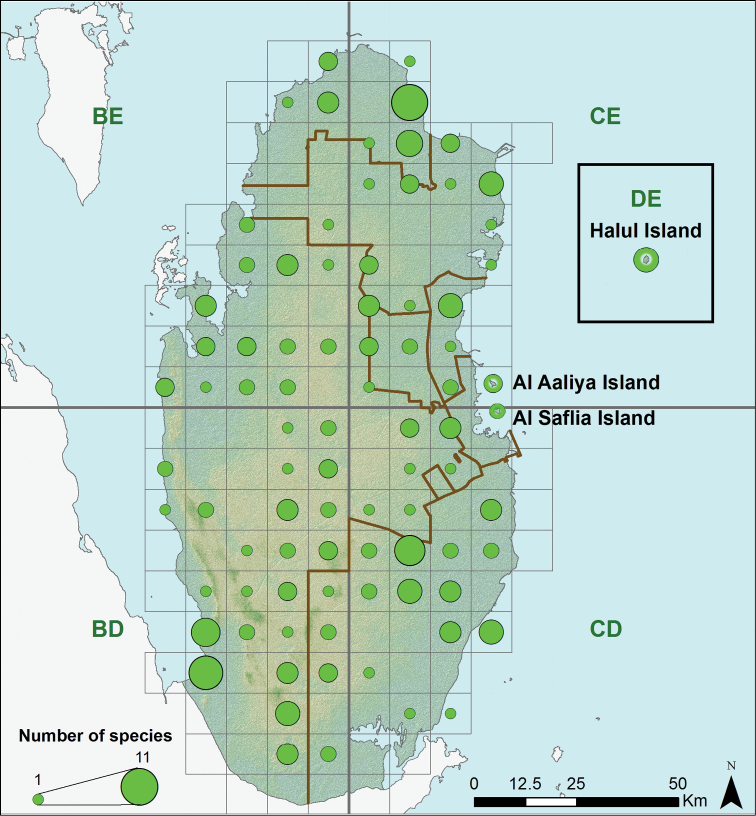
Lizard species richness at a resolution of 100 km^2^.

**Figure 5.**
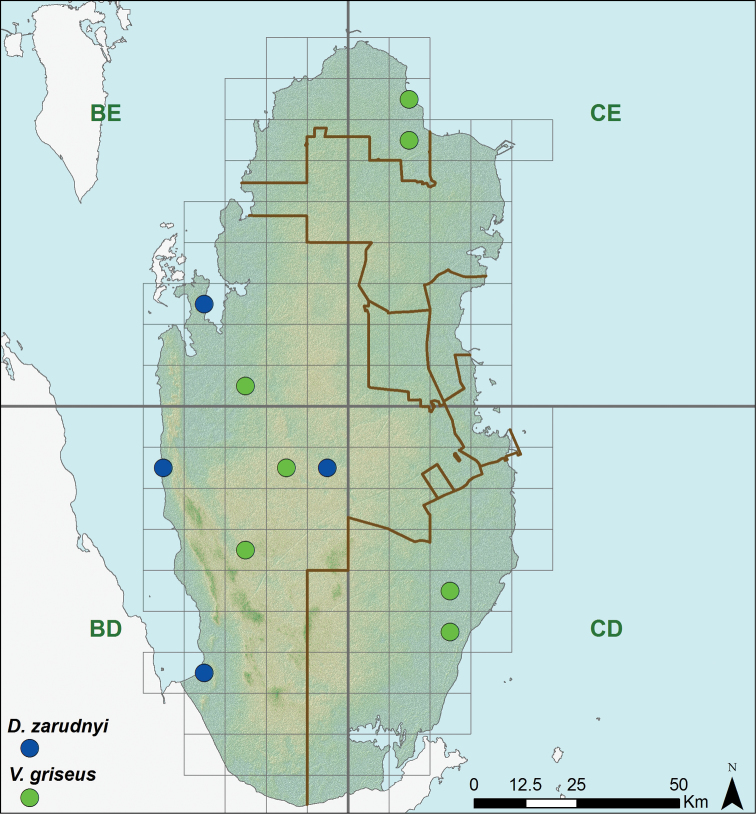
Distribution of *Diplometopon zarudnyi* and *Varanus griseus*.

**Figure 6.**
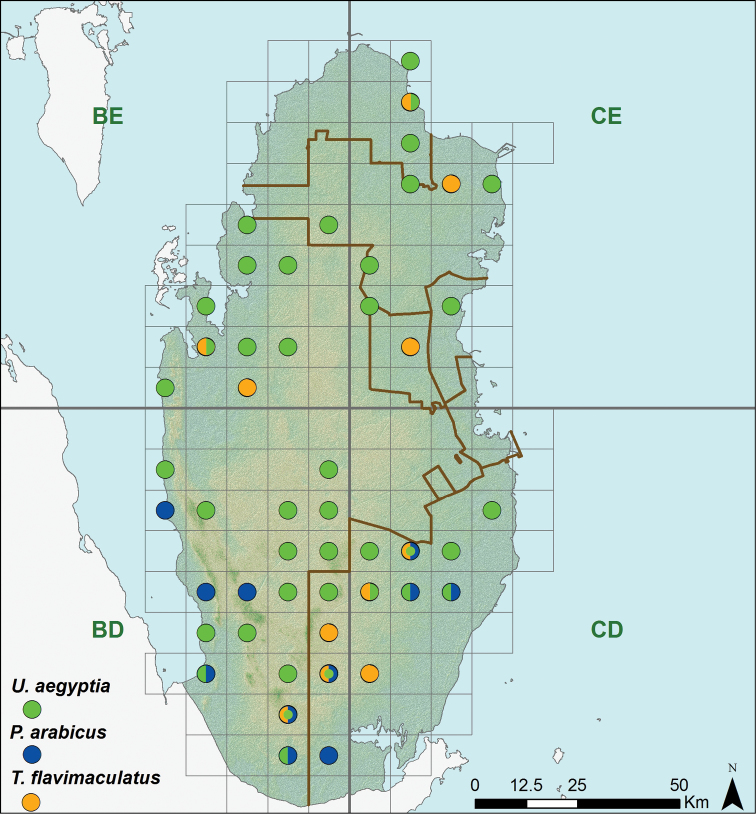
Distribution of three agamid species (*Phrynocephalus arabicus*, *Trapelus flavimaculatus*, *Uromastyx aegyptia*).

**Figure 7.**
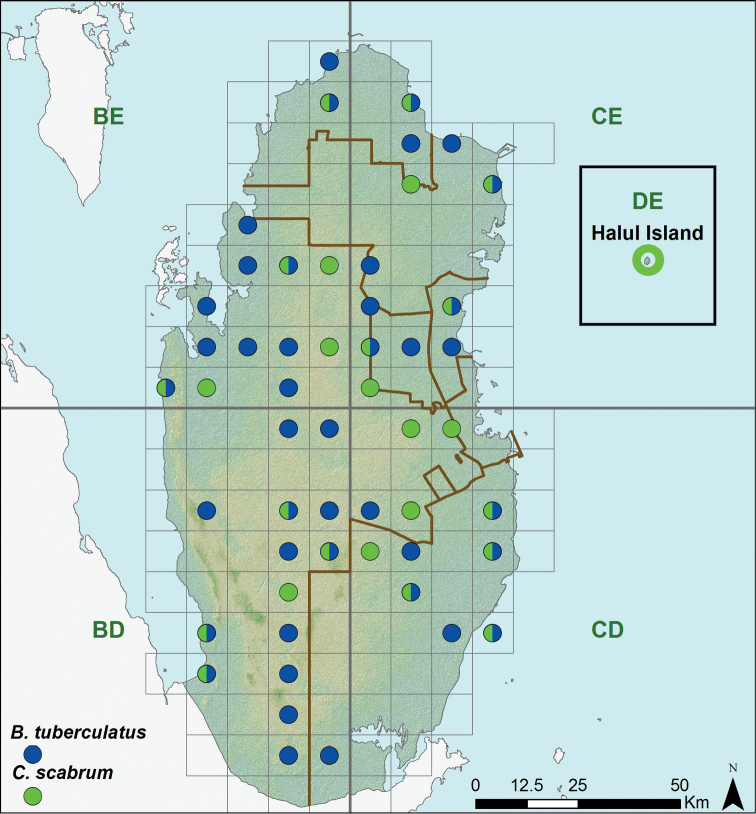
Distribution of *Bunopus tuberculatus* and *Cyrtopodion scabrum*.

**Figure 8.**
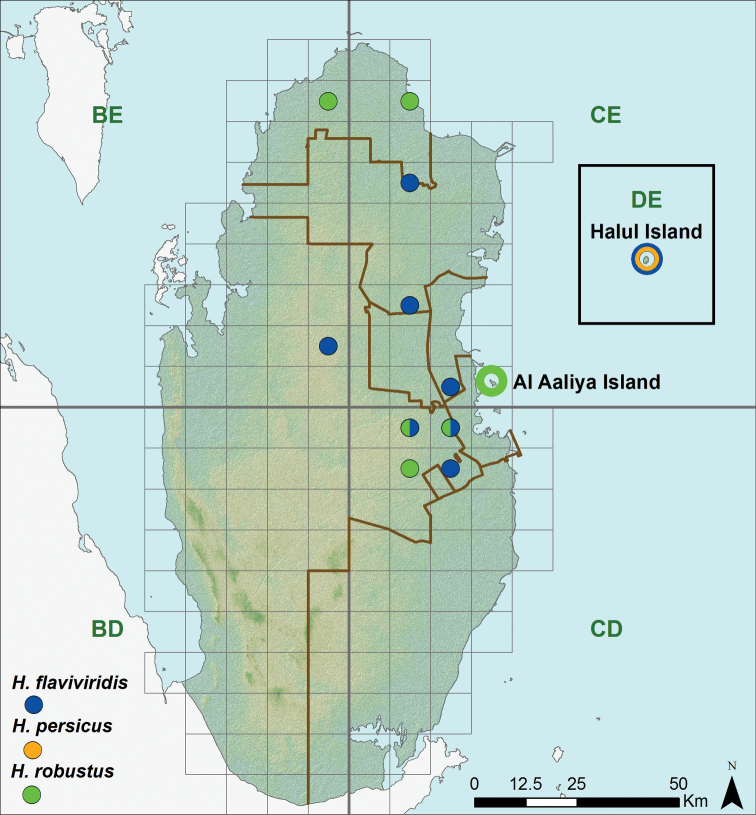
Distribution of the species of the genus *Hemidactylus*.

**Figure 9.**
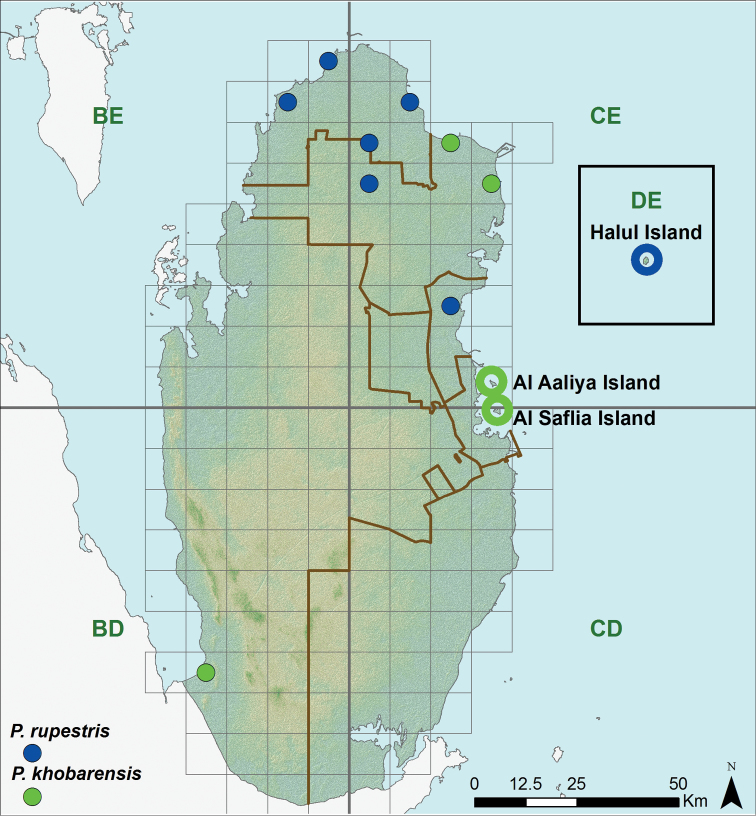
Distribution of *Pseudoceramodactylus khobarensis* and *Pristurus rupestris*.

**Figure 10.**
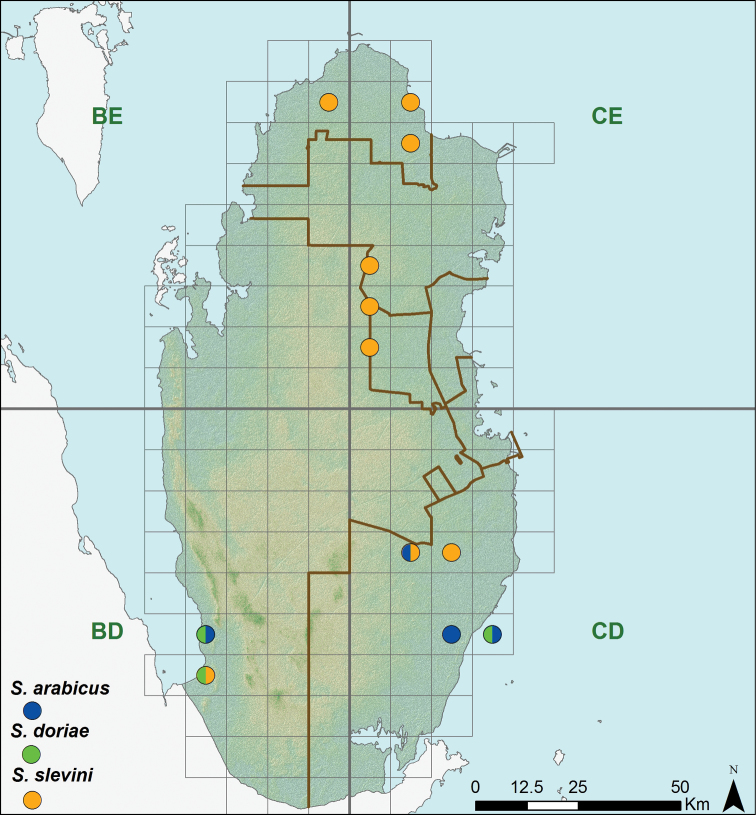
Distribution of the species of the genus *Stenodactylus*.

**Figure 11.**
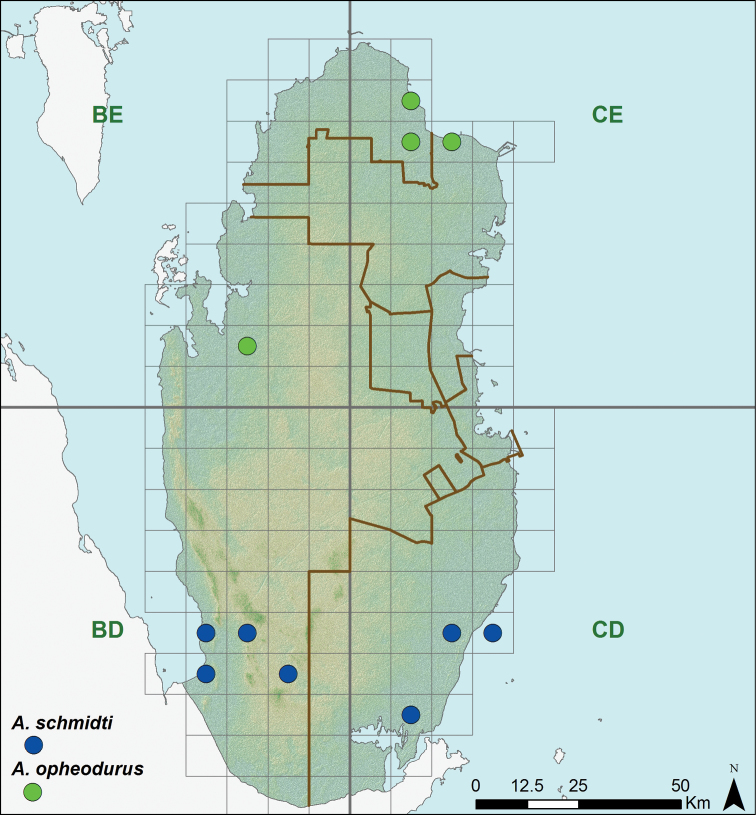
Distribution of the species of the genus *Acanthodactylus*.

**Figure 12.**
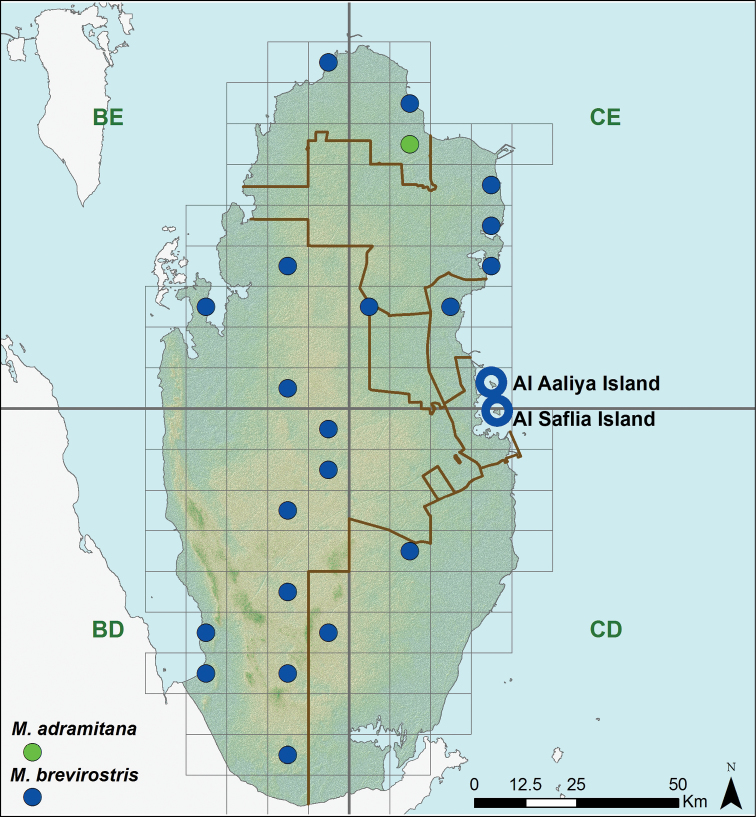
Distribution of the species of the genus *Mesalina*.

**Figure 13.**
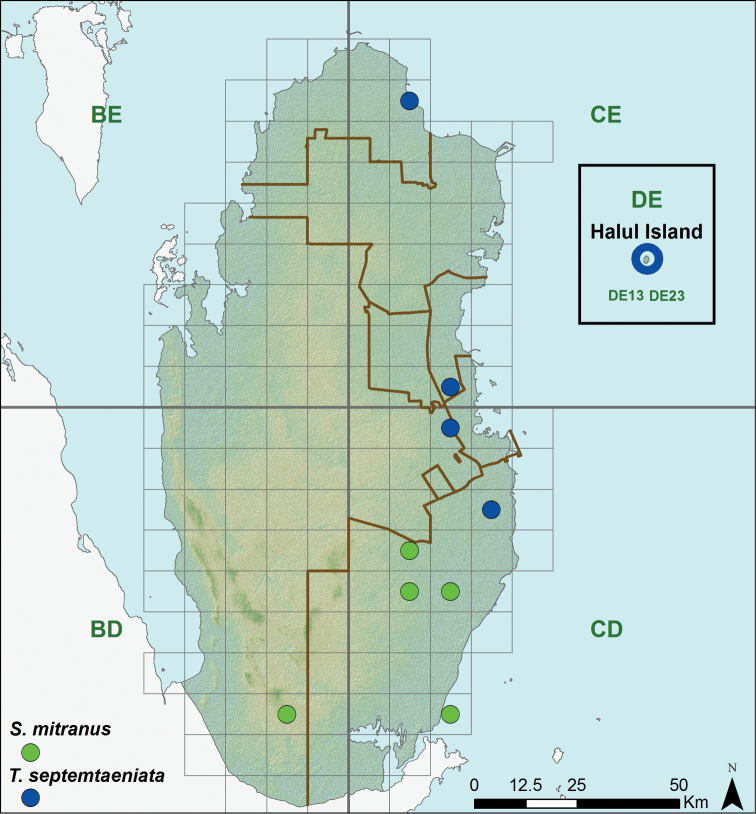
Distribution of Scincidae species (*Scincus mitranus* and *Trachylepis septemtaeniata*).

**Figure 14.**
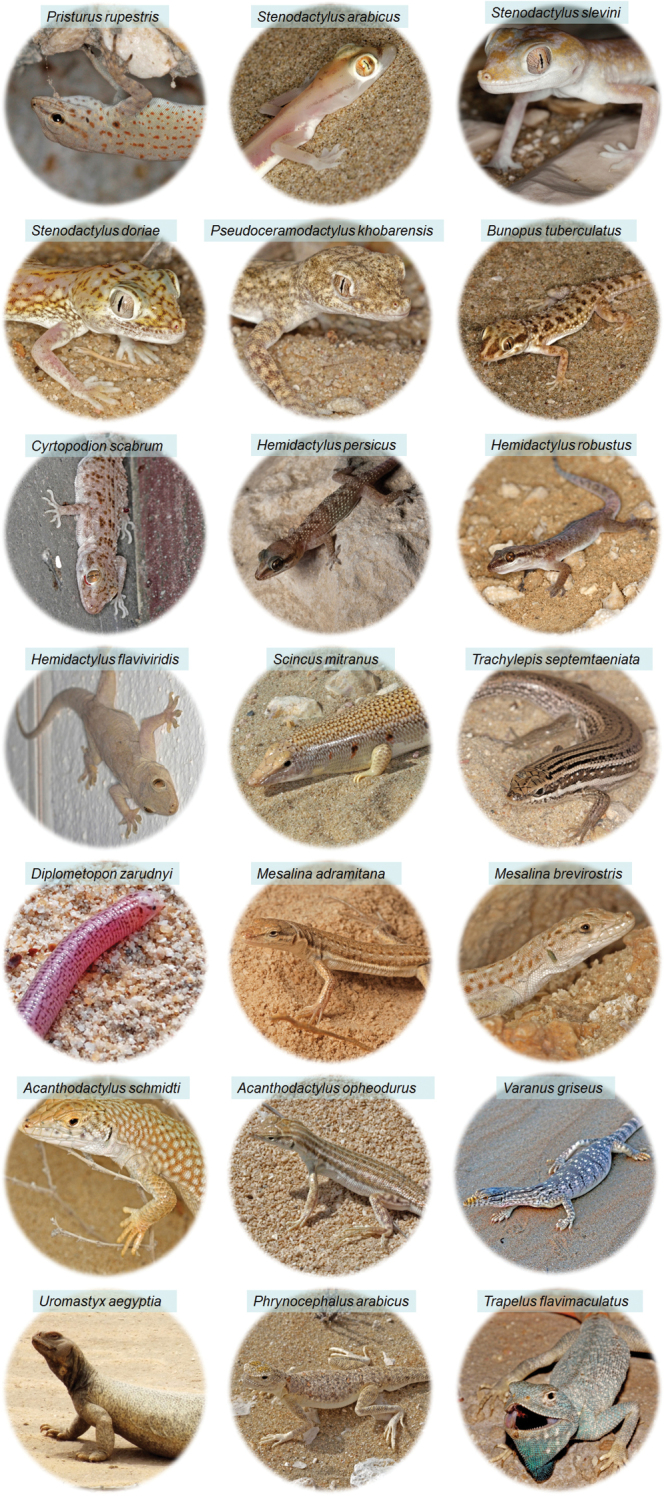
Photos of lizard species inventoried in Qatar (Author: Valdeón A, except for *Diplometopon zarudnyi* (Yamaguchi N)).

We inventoried 21 species of lizards belonging to seven families: Gekkonidae with nine species, Lacertidae with four species, Agamidae with three, Scincidae with two and Varanidae, Trogonophiidae and Sphaerodactylidae with one species each (Table 1). The number of lizard species observed per day ranged between 1 to 10 (average of 4.1 ± 2.1). In the island Al Saflia we only found two species (*Pseudoceramodactylus khobarensis* and *Mesalina brevirostris*), in Al Aaliya island we found three species (*Pseudoceramodactylus khobarensis*, *Mesalina brevirostris* and *Hemidactylus robustus*), and in Halul island we found five species (*Hemidactylus persicus*, *Hemidactylus flaviviridis*, *Cyrtopodion scabrum*, *Pristurus rupestris* and *Trachylepis septemtaeniata*). Based on our measurements of relative abundance, we found that four species of lizards appear to be the most abundant: *Bunopus tuberculatus*, *Cyrtopodion scabrum*, *Uromastyx aegyptia* and *Mesalina brevirostris* (Figure 2). The species accumulation curve approaches a plateau, suggesting that the majority of species present has been inventoried (Figure 3). The use of non-parametric estimators of species richness supports this statement indicating that only 2–4 species of lizards remain to be discovered (estimator, mean value ± standard deviation): ICE 23.61 ± 0.01, Chao 2 25.83 ± 5.86, Jackknife 1 25.83 ± 2.01, Bootstrap 23.11, and Michaelis-Menten 24.63. The preliminary lizard species richness varied largely across Qatar between 1–11 species per grid square (Figure 4). The distribution maps for the 21 lizard species in Qatar are presented in Figures 5–13, and Figure 14 shows the photos of the lizards.

**Table 1.** Lizard species inventoried in Qatar during the surveys conducted in 2012–2013, and publications where the species (or other species probably mistaken with correct species) are recorded for the first time in Qatar.

Species	Family	First record in Qatar
*Pristurus rupestris* Blanford, 1874 (a)	Sphaerodactylidae	Ministry of Environment 2004
*Pseudoceramodactylus khobarensis* Haas, 1957	Gekkonidae	Valdeón et al. 2013a
*Stenodactylus arabicus* (Haas, 1957)	Gekkonidae	Metallinou et al. 2012
*Stenodactylus doriae* (Blanford, 1874) (a)	Gekkonidae	Metallinou et al. 2012
*Stenodactylus slevini* Haas, 1957	Gekkonidae	Arnold 1980b
*Bunopus tuberculatus* Blanford, 1874 (a)	Gekkonidae	Mohammed 1988
*Cyrtopodion scabrum* (Heyden, 1827)	Gekkonidae	Mohammed 1988 (cited as *Gymnodactylus scaber*)
*Hemidactylus flaviviridis* Rüppell, 1835	Gekkonidae	Mohammed 1988
*Hemidactylus persicus* Anderson, 1872	Gekkonidae	Castilla et al. 2013
*Hemidactylus robustus* Heyden, 1827	Gekkonidae	Mohammed 1988 (cited as *Hemidactylus turcicus*)
*Scincus mitranus* Anderson, 1871	Scincidae	Mohammed 1988 (cited *Scincus scincus*, probably mistaken with *Scincus mitranus*)
*Trachylepis septemtaeniata* (Reuss, 1834)	Scincidae	Mohammed 1988 (cited as *Mabuya aurata*)
*Diplometopon zarudnyi* Nikolsky, 1907	Trogonophiidae	Mohammed 1988
*Mesalina adramitana* (Boulenger, 1917)	Lacertidae	Arnold 1980a
*Mesalina brevirostris* Blanford, 1874 (b)	Lacertidae	Mohammed 1988 (cited as *Eremias brevirostris*)
*Acanthodactylus schmidti* Haas, 1957	Lacertidae	Mohammed 1988 (cited *Acanthodactylus scutellatus*, probably mistaken with *Acanthodactylus schmidti*)
*Acanthodactylus opheodurus* Arnold, 1980	Lacertidae	Mohammed 1988 (cited *Acanthodactylus boskianus*, probably mistaken with *Acanthodactylus opheodurus*)
*Varanus griseus* (Daudin, 1803)	Varanidae	Mohammed 1988
*Uromastyx aegyptia* (Forskål, 1775)	Agamidae	Mohammed 1988 (cited as *Uromastyx microlepis*)
*Phrynocephalus arabicus* Anderson, 1894	Agamidae	Mohammed 1988 (cited as *Phrynocephalus nejdensis*)
*Trapelus flavimaculatus* Rüppell, 1835	Agamidae	Mohammed 1988 (cited as *Agama flavimaculata*)

## Discussion

In this study we provide the first consistent list of lizard species for Qatar, and the first distribution maps based on field surveys. Our study fills a gap in the knowledge of lizard distributions in the Arabian Peninsula and in Qatar specifically. Except for the study of Mohammed (1988), previous data has not been systematic and only included anecdotal references (Arnold 1980b, Leviton et al. 1992, Metallinou et al. 2012, Sindaco and Jeremčenko 2008).

During our surveys we attempted to collect data throughout the entire country; however this was not possible due to difficulties of accessing certain areas (e.g. industrial and private properties). The SE part of Qatar is difficult to access due to sandy soils and that was not fully sampled either. There are also differences in lizard species detectability due to differences in body size (e.g. maximum snout-vent length in *Uromastyx aegyptia* of 375 mm and only 32 mm in *Pristurus rupestris* (Meiri 2008)), behavior and period of activity (night or day). The lack of past information on the lizard species present in Qatar does not allow identifying allochtonous from native species. We consider species strictly associated with urban areas (e.g., *Hemidactylus flaviviridis*) as probably introduced, but further phylogeographic studies are required to elucidate their status. However, despite such difficulties, our lizard species inventory seems to be nearly completed as indicated by the estimators of species richness and the SAC (Fig. 3). Nevertheless, it could be not surprising to find in Qatar additional lizard species that occur in the vicinity of Qatar. The maps provided in Sindaco and Jeremčenko (2008) show that 13 lizard species are present near Qatar, including *Ptyodactylus hasselquistii*, *Teratoscincus (scincus) keyserlingii*, *Phrynocephalus maculatus*, *Pseudotrapelus* sp., *Trapelus ruderatus*, *Trapelus pallidus*, *Chalcides ocellatus*, *Scincus scincus*, *Acanthodactylus boskianus*, *Acanthodactylus haasi*, *Acanthodactylus gongrorhynchatus*, *Acanthodactylus scutellatus*, and *Mesalina guttulata*.

The preliminary species distribution maps provided in this study allow, nevertheless, for further analysis on distribution patterns of abundance, rarity, richness and assemblage composition at larger spatial scales (Elith et al. 2010, Baselga et al. 2012). The distribution data are also valuable for conservation planning and modeling species distribution at regional and global level (Sillero et al. 2005).

The reptiles of Qatar are threatened by the rapid human population growth that increased from several tens of thousand inhabitants to almost two million in the last century, habitat destruction caused by construction development, gas and oil extraction and transport facilities, and the introduction of alien species (especially cats). The human impact is unevenly distributed, with high human impact in and around Doha, moderate along the coast and low in the interior (WCS 2005). Qatar produces around 2 million tons of solid municipal waste annually, corresponding to a daily generation rate of about 2.5 kg per capita that are disposed mainly through landfill and composting. This increase in solid waste generation not only results in the environmental pollution but also habitat destruction (Al-Maaded et al. 2012). There are 7790 km of roads (0.67 km road/km^2^), of which 90% are paved, and 571 cars per 1,000 people (2002 est) (Hutchinson Encyclopedia 2011). High traffic on roads, particularly in rural areas, can result in high mortality in reptile populations (e.g. Shepard et al. 2008). Road-kills are frequent and while it is difficult to document it in small lizards, carcasses of larger lizards like *Uromastyx* are often found along roads. The response of lizards to the relatively recent human impact in Qatar provides an excellent example of the winner-loser concept (McKinney and Lockwood 1999), with several species benefiting from man-made artificial habitats (e.g. *Cyrtopodion scabrum*, *Hemidactylus flaviviridis*, *Hemidactylus robustus*). We estimate that human activities will result in changes in the ranges of the lizards of Qatar and stress the urgent need for a complete species inventory and mapping, as a background study for a future monitoring program.

## Conclusion

The distribution maps presented in this paper as visualized occurrence records fill a gap in the knowledge of biodiversity in Qatar, and will help the prioritization of conservation efforts and the identification of important conservation areas for lizards. This study is a first step in updating the Qatar databases of lizards and wildlife, with the goal to make scientific biodiversity data available and useful for the international community.

## References

[B1] Al-Maaded M Madi NK Kahraman R Hodzic A Ozerkan NG (2012) An Overview of Solid Waste Management and Plastic Recycling in Qatar. J Polym Environ 20: 186–194. doi: 10.1007/s10924-011-0332-2

[B2] Anonymous (2010) Amphisbaenid record – another first for Qatar? Qatar Natural History Group 3: 16.

[B3] Anderson J (1871) Description of a new species of *Scincus*. P. Asiat. Soc. Bengal 1871: 115–116.

[B4] Anderson J (1872) On some Persian, Himalayan and other Reptiles. Proc. Zool. Soc. London 1872: 371–404.

[B5] Anderson J (1894) On two new species of agamoid lizards from the Hardramut, South-Eastern Arabia. Ann. Mag. Nat. Hist. 6th Series 14: 376–378.

[B6] Arnold EN (1980a) The reptiles and amphibians of Dhofar, southern Arabia. Journal of Oman Studies Special Report (No. 2) 1980: 273–332.

[B7] Arnold EN (1980b) Reptiles of Saudi Arabia. A review of the lizard genus *Stenodactylus* (Reptilia: Gekkonidae). Fauna of Saudi Arabia 2: 368–404.

[B8] Arnold EN (1986) A Key and Annotated Check List to the Lizards and Amphisbaenians of Arabia. Fauna of Saudi Arabia 8: 385–435.

[B9] Arnold EN (1995) Atlas of Amphibians and Reptiles in Britain. ITE research publication no. 10 Centre for Ecology and Hydrology. Natural Environment Research Council HMSO, London, 42 pp.

[B10] Baselga A Lobo JM Svenning JC Araújo MB (2012) Global patterns in the shape of species geographical ranges reveal range determinants. Journal of Biogeography 39: 760–771. doi: 10.1111/j.1365-2699.2011.02612.x

[B11] Batanouny KH (1981) Ecology and flora of Qatar. Environmental Studies Center, University of Qatar, 245 pp.

[B12] Bauer A Masroor R Titus-McQuillan J Heinicke MP Heinicke MP Daza JD Jackman, TR (2013) A preliminary phylogeny of the Palearctic naked-toed geckos (Reptilia: Squamata: Gekkonidae) with taxonomic implications. Zootaxa 3599: 301–324. doi: 10.11646/zootaxa.3599.4.1 24613954

[B13] Blanford WT (1874a) Descriptions of new lizards from Persia and Baluchistan. Ann. Mag. Nat. Hist. 4th Series 13: 453–455.

[B14] Blanford WT (1874b) Descriptions of new Reptilia and Amphibia from Persia and Baluchistan. Ann. Mag. Nat. Hist. 4th Series 14: 31–35.

[B15] Böhm M Collen B Baillie JEM Bowles P Chanson J Cox N Hammerson G Hoffmann M Livingstone SR Ram M Rhodin AGJ Stuart SN van Dijk PP Young BE Afuang LE Aghasyan A García A Aguilar C Ajtic R Akarsu F Alencar LRV Allison A Ananjeva N Anderson S Andrén C Ariano-Sánchez D Arredondo JC Auliya M Austin CC Avci A Baker PJ Barreto-Lima AF Barrio-Amorós CL Basu D Bates MF Batistella A Bauer A Bennett D Böhme W Broadley D Brown R Burgess J Captain A Carreira S Castañeda MDR Castro F Catenazzi A Cedeño-Vázquez JR Chapple DG Cheylan M Cisneros-Heredia DF Cogălniceanu D Cogger H Corti C Costa GC Couper PJ Courtney T Crnobrnja-Isailovic J Crochet PA Crother B Cruz F Daltry JC Daniels RJR Das I de Silva A Diesmos AC Dirksen L Doan TM Dodd CK Doody JS Dorcas ME Duarte de Barros Filho J Egan VT El Mouden EH Embert D Espinoza RE Fallabrino A Feng X Feng ZJ Fitzgerald L Flores-Villela O França FGR Frost D Gadsden H Gamble T Ganesh SR Garcia MA García-Pérez JE Gatus J Gaulke M Geniez P Georges A Gerlach J Goldberg S Gonzalez JCT Gower DJ Grant T Greenbaum E Grieco C Guo P Hamilton AM Hare K Hedges SB Heideman N Hilton-Taylor C Hitchmough R Hollingsworth B Hutchinson M Ineich I Iverson J Jaksic FM Jenkins R Joger U Jose R Kaska Y Kaya U Keogh JS Köhler G Kuchling G Kumlutaş Y Kwet A La Marca E Lamar W Lane A Lardner B Latta C Latta G Lau M Lavin P Lawson D LeBreton M Lehr E Limpus D Lipczynski N Lobo AS López-Luna MA Luiselli L Lukoschek V Lundberg M Lymberakis P Macey R Magnusson WE Mahler DL Malhotra A Mariaux J Maritz B Marques OAV Márquez R Martins M Masterson G Mateo JA Mathew R Mathews N Mayer G McCranie JR Measey GJ Mendoza-Quijano F Menegon M Métrailler S Milton DA Montgomery C Morato SAA Mott T Muñoz-Alonso A Murphy J Nguyen TQ Nilson G Nogueira C Núñez H Orlov N Ota H Ottenwalder J Papenfuss T Pasachnik S Passos P Pauwels OSG Pérez-Buitrago N Pérez-Mellado V Pianka ER Pleguezuelos J Pollock C Ponce-Campos P Powell R Pupin F Quintero Díaz GE Radder R Ramer J Rasmussen AR Raxworthy C Reynolds R Richman N Rico EL Riservato E Rivas G da Rocha PLB Rödel MO Rodríguez Schettino L Roosenburg WM Ross JP Sadek R Sanders K Santos-Barrera G Schleich HH Schmidt BR Schmitz A Sharifi M Shea G Shi HT Shine R Sindaco R Slimani T Somaweera R Spawls S Stafford P Stuebing R Sweet S Sy E Temple HJ Tognelli MF Tolley K Tolson PJ Tuniyev B Tuniyev S üzüm N van Buurt G Van Sluys M Velasco A Vences M Veselý M Vinke S Vinke T Vogel G Vogrin M Vogt RC Wearn OR Werner YL Whiting MJ Wiewandt T Wilkinson J Wilson B Wren S Zamin T Zhou K Zug G (2013) The conservation status of the world’s reptiles. Biological Conservation 157: 372–385. doi: 10.1016/j.biocon.2012.07.015

[B16] Boulenger GA (1917) Descriptions of new lizards of the family Lacertidae. Ann. Mag. Nat. Hist. 8th Series 19: 277–279.

[B17] Castilla AM Richer R Herrel A Conkey AAT Tribuna J Al-Thani M (2011a) First evidence of scavenging behaviour in the herbivorous lizard *Uromastyx aegyptia microlepis*. Journal of Arid Environments 75: 671–673. doi: 10.1016/j.jaridenv.2011.02.005

[B18] Castilla AM Richer R Herrel A Conkey AAT Tribuna J Chan R Martínez de Aragón J Böer B Mohtar R (2011b) Plant diversity in the diet of the lizard *Uromastyx aegyptia microlepis* in Qatar: The effect of zone, sampling date and faeces size. Proceedings of the Qatar Foundation Annual Research Forum 2011a (QF-ARF). doi: 10.5339/qfarf.2011.evp7

[B19] Castilla AM Valdeón A Cogălniceanu D Gosá A Alkuwary A Saifelnasr EH Al Naimi S Al-Hemaidi AA (2013) First record of a gecko species to the fauna of Qatar: *Hemidactylus persicus* Anderson, 1872 (Gekkonidae). QScience Connect 2013.28. doi: 10.5339/connect.2013.28

[B20] Colwell RK (2013) EstimateS: Statistical estimation of species richness and shared species from samples. Version 9. User’s Guide and application published at: http://purl.oclc.org/estimates

[B21] Cox NA Temple HJ (2009) European red list of reptiles. Office for Official Publications of the European Communities, Luxembourg, 32 pp.

[B22] Daudin FM (1803) Histoire Naturelle, Générale et Particulière des Reptiles. Vol. 8 F. Dufart, Paris, 442 pp.

[B23] Elith J Kearney M Phillips S (2010) The art of modelling range‐shifting species. Methods in Ecology and Evolution 1: 330–342. doi: 10.1111/j.2041-210X.2010.00036.x

[B24] ESA European Space Agency (2010) GlobCover 2009 v2.3. http://due.esrin.esa.int/globcover/

[B25] Forskål P (1775) Descriptiones animalium, avium, amphibiorum, piscium, insectorum, vermium; quae in itinere Orientali observavit Petrus Forskål. Mölleri, Hauniae, xxxiv + 164 pp.

[B26] Fujita MK Papenfuss TJ (2011) Molecular systematics of *Stenodactylus* (Gekkonidae), an Afro-Arabian gecko species complex. Molecular Phylogenetics and Evolution 58: 71–75. doi: 10.1016/j.ympev.2010.10.014 21035555

[B27] Gamble T Bauer AM Greenbaum E Jackman TR (2008) Evidence for Gondwanan vicariance in an ancient clade of gecko lizards. Journal of Biogeography 35: 88–104. doi: 10.1111/j.1365-2699.2007.01770.x

[B28] Gibbons J Scott DE Ryan TJ Buhlmann KA Tuberville TD Metts BS Greene JL Mills T Leiden Y Poppy S Wiine C (2000) The Global Decline of Reptiles, *Déjà Vu* Amphibians. BioScience 50: 653–666. doi: 10.1641/0006-3568(2000)050[0653:TGDORD]2.0.CO;2

[B29] Godinho R Teixeira J Rebelo R Segurado P Loureiro A Álvares F Gomes N Cardoso P Camilo-Alves C Brito JC (1999) Atlas of the continental Portuguese herpetofauna: an assemblage of published data. Revista Española de Herpetología 13: 61–82.

[B30] Gosá A Bergerandi A (1994) Atlas de distribución de los Anfibios y Reptiles de Navarra. Munibe (Ciencias Naturales) 46: 109–189.

[B31] Haas G (1957) Some amphibians and reptiles from Arabia. Proc. Cal. Acad. Sci. 29(3): 47–86.

[B32] Herrel A Castilla AM Al-Sulaiti M Wessels J (2013) Does large body size relax constraints on bite force generation in lizards of the genus *Uromastyx*. Journal of Zoology. doi: 10.1111/jzo.12089

[B33] Heyden CHG von (1827) Reptilien. In: Rüppell E Atlas zu Reise im nördlichen Afrika. l. Zoologie. HL Brönner, Frankfurt a.M., 1–24.

[B34] Hutchinson Encyclopedia (2011) Qatar. eLibrary. Web. 06 Nov. 2013.

[B35] Leviton AE Anderson SC Adler K Minton SA (1992) Handbook to Middle East Amphibians and Reptiles. Society for the Study of Amphibians and Reptiles Oxford, USA, 252 pp.

[B36] Martín J Castilla AM López P Al Jaidah M Mohtar R (2012) Lipophilic compounds in femoral gland secretions of spiny-tailed lizard, dhub, *Uromastyx aegyptia microlepis* (Reptilia, Agamidae) from the Qatar desert. Proceedings of the Qatar Foundation Annual Research Forum 2012 (QF-ARF). doi: 10.5339/qfarf.2012.EEP53

[B37] Matin S Chitale VS Behera MD Mishra B Roy PS (2012) Fauna data integration and species distribution modelling as two major advantages of geoinformatics-based phytobiodiversity study in today’s fast changing climate. Biodiversity and Conservation 21: 1229–1250. doi: 10.1007/s10531-012-0233-2

[B38] McDiarmid RW Foster MS Guyer C Gibbons WJ Chernoff N (2012) Reptile Biodiversity. Standard methods for inventory and monitoring. University of California Press.

[B39] McKinney ML Lockwood JL (1999) Biotic homogenization: a few winners replacing many losers in the next mass extinction. Trends in Ecology and Evolution 14: 450–453. doi: 10.1016/S0169-5347(99)01679-1 10511724

[B40] Meiri S (2008) Evolution and ecology of lizard body sizes. Global Ecology and Biogeography 17: 724–734. doi: 10.1111/j.1466-8238.2008.00414.x

[B41] Metallinou M Arnold EN Crochet P Geniez P Brito JC Lymberakis P Baha El Din S Sindaco R Robinson M Carranza S (2012) Conquering the Sahara and Arabian deserts: Systematics and biogeography of *Stenodactylus* geckos (Reptilia: Gekkonidae). BMC Evolutionary Biology 12: 258. doi: 10.1186/1471-2148-12-258 23273581 PMC3582542

[B42] Mohammed MBH (1988) Survey of the reptiles of Qatar. Proceedings of the Zoological Society of the Arab Republic Egypt 15: 17–26.

[B43] Moravec J Franzen M Böhme W (2006) Taxonomy, nomenclature and distribution of the *Trachylepis* (formerly *Mabuya*) *aurata* (Linnaeus, 1758) complex. Herpetologia Bonnensis II. Proceedings of the 13th Congress of the Societas Europaea Herpetologica, 89–93.

[B44] MOE Ministry of the Environment (2004) Qatar National Biodiversity Strategy and Action Plan Assessment. Biodiversity Inventory, Final Report. Ministry of the Environment, Doha, Qatar.

[B45] Nasher AK Al Thani RF Altaeb AA (2009) The University Farm, a potential field station for scientific research. Qatar Biodiversity Newsletter 13: 2–11.

[B46] NGA (2013) DMA Technical Manual 8358.1. http://earth-info.nga.mil/GandG/publications/tm8358.1/toc.html

[B47] Nikolsky AM (1907) Reptiles et amphibiens recueillis (part.) M. N. A. Zarudny en Perse en 1903–1904. Ann. Mus. Zool. Acad. Imp. Sci., St. Petersburg 10 [1905]: 260–301. [in Russian and Latin]

[B48] Qatar Statistics Authority (2013) Qatar Atlas, Doha, Qatar, 1–303.

[B49] Oldham MJ Weller WF (2000) Ontario Herpetofaunal Atlas. Natural Heritage Information Centre, Ontario Ministry of Natural Resources.

[B50] Pickard CR Towns DR (1988) Atlas of the amphibians and reptiles of New Zealand. Conservation Sciences Publication Number 1. Science and Research Directorate. Department of Conservation, Wellington, 59 pp.

[B51] Pleguezuelos JM Márquez R Lizana M (2002) Atlas y Libro Rojo de los Anfibios y Reptiles de España. Dirección General de Conservación de la Naturaleza – Asociación Herpetológica Española (2ª impresión), Madrid, 587 pp.

[B52] Primack R (2010) Essentials of Conservation Biology, 5th edition Sinauer Associates, Sunderland, MA, 601 pp.

[B53] Pyron RA Burbrink FT Wiens JJ (2013) A phylogeny and revised classification of Squamata, including 4161 species of lizards and snakes. BMC Evolutionary Biology 13: 93. doi: 10.1186/1471-2148-13-93 23627680 PMC3682911

[B54] Reese GC Wilson KR Hoeting JA Flather CH (2005) Factors affecting species distribution predictions: a simulation modeling experiment. Ecological Applications 15: 554–564. doi: 10.1890/03-5374

[B55] Reuss A (1834) Zoologische Miscellen, Reptilien. Abhandlungen aus dem Gebiete der beschreibenden Naturgeschichte. Museum Senckenbergianum, Frankfurt am Main 1(6): 27–62.

[B56] Richer R (2008) Conservation in Qatar. Impacts of increasing industrialization. CIRS. Centre for International and Regional Studies. Georgetown University School of Foreign Service in Qatar, 27 pp.

[B57] Rüppell E (1835) Neue Wirbelthiere zu der Fauna von Abyssinien gehörig, entdeckt und beschrieben. Amphibien. S. Schmerber, Frankfurt a.M., 437 pp.

[B58] Supreme Council for Environment and Natural Reserves (SCENR) (2007) Protected Area Action Plan 2008–2013 for Qatar. Department of Wildlife Conservation, 25 pp.

[B59] Sillero N Celaya L Martin-Alfageme S (2005) Using Geographical Information System (GIS) to make an atlas: a proposal to collect, store, map and analyse chorological data for herpetofauna. Revista Española de Herpetología 19: 87–101.

[B60] Sindaco R Jeremčenko VK (2008) The Reptiles of the Western Palearctic. 1. Annotated checklist and distributional atlas of the turtles, crocodiles, amphisbaenians and lizards of Europe, North Africa, Middle East and Central Asia. Edizione Belvedere, Latina.

[B61] Shepard DB Dreslik MJ Jellen BC Phillips CA (2008) Reptile Road Mortality around an Oasis in the Illinois Corn Desert with Emphasis on the Endangered Eastern Massasauga. Copeia 2008: 350–359. doi: 10.1643/CE-06-276

[B62] Underwood G (1954) On the classification and evolution of geckos. Proceedings of the Zoological Society of London 124: 469–492. doi: 10.1111/j.1469-7998.1954.tb07789.x

[B63] UPDA Urban Planning & Development Authority (2009) Qatar Survey Manual. Urban Planning & Development Authority, Doha, Qatar.

[B64] Valdeón A Castilla AM Cogălniceanu D Gosá A Alkuwary A Saifelnasr E Naumann E Mas-Peinado P Richer R Al-Hemaidi A (2013a) On the presence and distribution of the Gulf sand gecko, *Pseudoceramodactylus khobarensis* Haas, 1957 (Reptilia: Squamata: Gekkonidae) in Qatar. QScience Connect 2013.34. doi: 10.5339/connect.2013.34

[B65] Valdeón A Castilla AM Laso B Longares LA Bukhari S Mohielden Y Mohd Al-Hemaidi AA (2013b) Development of a Qatar National Biodiversity Grid (QNBG) to create Biodiversity Atlas in Qatar. Proceedings of the Qatar Foundation Annual Research Conference 2013 (QF-ARC). doi: 10.5339/qfarf.2013.EESP-040

[B66] WCS Wildlife Conservation Society - WCS Center for International Earth Science Information Network- CIESIN - Columbia University (2005) Last of the Wild Project, Version 2, 2005 (LWP-2): Global Human Footprint Dataset (Geographic). NASA Socioeconomic Data and Applications Center (SEDAC), Palisades, NY http://sedac.ciesin.columbia.edu/data/set/wildareas-v2-human-footprint-geographic [accessed 11 November 2013]

